# More Surprises in the Development of an HIV Vaccine

**DOI:** 10.3389/fimmu.2014.00329

**Published:** 2014-07-14

**Authors:** José Esparza, Marc H. V. Van Regenmortel

**Affiliations:** ^1^Institute of Human Virology, University of Maryland School of Medicine, Baltimore, MD, USA; ^2^CNRS, UMR7242-Institut de Recherche de l’Ecole de Biotechnologie de Strasbourg (IREBS), Université de Strasbourg, Illkirch-Graffenstaden, France

**Keywords:** inactivated SIV, bacterial adjuvants, lactobacilli, SIV vaccine, HIV vaccine, tolerogenic vaccine

In the current issue of Frontiers in Immunology, Jean-Marie Andrieu and collaborators, report results from non-human primate experiments designed to explore a new vaccine concept aimed at inducing tolerance to the simian immunodeficiency virus (SIV) (1). This approach, which is significantly different from other vaccine concepts tested to date, resulted in a surprisingly high level of protection. If the results are confirmed and extended to the human immunodeficiency virus (HIV), this approach may represent a game changing strategy, which should be welcomed by a field that has been marred by mostly disappointing results.

When HIV was discovered and established as the cause of the Acquired Immune Deficiency Syndrome (AIDS) in 1983–1984, there was an expectation that a preventive vaccine would be rapidly developed (2).

Vaccines against several major human viral diseases (polio, measles, mumps, rubella, etc.) were successfully developed during the preceding two or three decades, mostly using live-attenuated viruses, and designed to induce the same type of protective immune responses that develop after natural infection. Moreover, recent advances in molecular biology and recombinant DNA technologies were offering exciting new opportunities for vaccine development, first achieved with the licensure in 1986 of a recombinant vaccine against hepatitis B (3, 4).

Since the use of whole-inactivated or of live-attenuated vaccines was considered too risky for a pathogen such as HIV, the molecular approach was the one selected by early HIV vaccine developers. That decision was also based on the confidence that new knowledge on the structure and function of the virus, as well as of the pathogenesis of the disease, will provide the information needed for the rational development of a much needed HIV vaccine (5).

In that environment of optimism, the first phase I clinical trials of HIV vaccines started in the United States in 1988. Since then, more than 200 clinical trials have been conducted globally, the majority of them phase I and II trials, to assess the safety and immunogenicity of different vaccine candidates. Those candidate vaccines were developed and tested according to prevailing paradigms that sequentially explored the role of neutralizing antibodies, cell-mediated immunity (CMI) and, more recently, other potential mechanisms of immune protection (2, 6).

Although much has been learned from those small-scale clinical trials, the results from phase IIb/III efficacy trials are the ones that have driven major changes on how HIV vaccine research is advanced. Those trials have also given us a few surprises. Fortunately, the field has been able to learn from those lessons and steadily move forward.

Perhaps the first major surprise was when in 1994 we learned that field isolates of HIV were more difficult to neutralize *in vitro* than laboratory-adapted strains, and that proposed existing candidate vaccines could not induce the appropriate type of neutralizing antibodies, a problem that we are still struggling to solve. Nevertheless, in the early 2000s, two gp120 candidate vaccines from VaxGen were tested in efficacy trials and, as many predicted, they failed to protect. That failure shifted the field to CMI vaccines and to the suggestion that perhaps the best that an HIV vaccine could do is to decrease virus load in vaccinated individuals who became infected (7). Unfortunately, the STEP study, which tested the CMI concept using an adenovirus 5 vector, and which was a favorite approach of the HIV vaccine community, was halted in 2007 because of lack of efficacy (8). That was a major surprise that led to calls to slow down clinical trials and to go back to basic science (9).

The next major surprise came in 2009, when the results from the Thai RV144 were announced. The trial, which evaluated a canarypox prime followed by a gp120 boost, was strongly opposed by some of the leading HIV vaccine scientists (10). Unexpectedly, the trial showed for the first time that prevention of HIV infection was achievable by an HIV vaccine (11). In a commentary authored by the late Norman Letvin (12), who himself expressed concerns about the conduct of the RV144 trial, he indicated that the findings were not expected based on preclinical studies and human immunogenicity data, concluding with the lapidary remark that “*we have learned to expect the unexpected in our efforts to generate an effective HIV vaccine*.”

Although the observed protection in RV144 was modest (31.2%), those results not only brought new optimism to the field, but also triggered a major collaborative effort to try to identify immune correlates of protection (13). In this regard, novel and more promising vaccines are being developed that may result in higher levels of protective efficacy, including the use of vectors based on adenovirus 26 (Ad26) and cytomegalovirus (CMV) (14, 15).

Another surprise came when a careful statistical analysis of the step study confirmed that vaccination in fact enhanced HIV acquisition among a subset of the volunteers (16), an observation that was also been made in the Phambili study conducted in South Africa using the same vaccine as in the step study (17). The most likely explanation of the observed enhancement is a specific immune activation induced by the adenovirus 5 vectored vaccines. Although the mechanism is poorly understood, it does not seem to be present with another adenovirus 5 vectored HIV vaccine (18), and it is not clear how relevant it could be to other vaccine approaches (19). Nevertheless, it is well-known that activation of CD4^+^ cells is important for HIV replication, which creates a dilemma for vaccinologists, who have to thread a compromise between the desire to induce strong vaccine responses and, at the same time, avoid the immune activation that may enhanced HIV acquisition. In this and other regards, HIV/AIDS is different from other viral diseases for which vaccines have been developed, because forces vaccine developers to explore mechanisms that nature has not developed, especially when dealing with chronic infections (20).

It is in this context that Jean-Marie Andrieu and collaborators report in this journal (1) additional results from an approach that they first reported in 2012 (21, 22).

The investigators used Chinese macaques to explore the concept that the induction of immune tolerance to SIV induces protective immunity in the absence of usual humoral or cellular immune responses. To achieve that goal, inactivated SIV was intragastrically administered together with living bacterial adjuvants (BCG, *Lactobacillus plantarum*, or *Lactobacillus rhamnosus*) with the goal of inducing tolerance to the SIV antigens. In a series of experiments, the investigators showed that their approach protected the experimental animals from mucosal and parenteral challenges. Vaccination neither elicit SIV-specific antibodies nor cytotoxic T-lymphocytes but induced a previously unrecognized population of non-cytolytic MHCIb/E-restricted CD8^+^ T regulatory cells that suppressed the activation of SIV positive CD4^+^ T-lymphocytes. Although the number of monkeys is relatively small, the levels of protection are impressive, with 23 out of 24 animals protected in one of the experiments, when animals were challenged 48 months after vaccination.

The 2012 publication from this group (21) had very little impact in the field, perhaps because it was received with a degree of skepticism. After all, 30 years of intense vaccine research had not resulted in a practical effective vaccine, although an HIV vaccine is sorely needed to bring the HIV epidemic under control. No stone should remain unturned in its search, and the approach reported in this journal should not be dismissed *a priori*. Instead, it should be carefully considered by other scientists and appropriately confirmed or refuted by additional research.

In order to accelerate the development of an HIV vaccine, one of us has proposed a number of actions, including the suggestion to establish a program of truly innovative research with protected funding to explore out-of-the-paradigm approaches, perhaps allocating to this program not <10% of the total HIV vaccine investment (23). Out-of-the-paradigm approaches, such as the one proposed by Andrieu et al., should be further explored (24).

Paraphrasing Dean K. Simonton (25), the University of California psychologist who has dedicated his professional life to the study of creativity: good science contributes ideas that are original and useful, and we have plenty of those in the HIV vaccine field. However, the solution to the HIV vaccine challenge will require genius which, according to Simonton, is characterized not only by originality and usefulness, but also by surprising results.

## Conflict of Interest Statement

The authors declare that the research was conducted in the absence of any commercial or financial relationships that could be construed as a potential conflict of interest.
